# Insights into the Pathobiology of GM1 Gangliosidosis from Single-Nucleus Transcriptomic Analysis of CNS Cells in a Mouse Model

**DOI:** 10.3390/ijms25179712

**Published:** 2024-09-08

**Authors:** Sichi Liu, Ting Xie, Yonglan Huang

**Affiliations:** Department of Guangzhou Newborn Screening Center, Guangzhou Women and Children’s Medical Center, Guangzhou Medical University, Guangzhou 510623, China; liusichi0@outlook.com (S.L.); xieting19900111@outlook.com (T.X.)

**Keywords:** GM1 gangliosidosis, single-nucleus RNA sequencing, brain, cellular heterogeneity, neurodegenerative diseases, gene expression

## Abstract

GM1 gangliosidosis is a lysosomal storage disorder characterized by the accumulation of GM1 ganglioside, leading to severe neurodegeneration and early mortality. The disease primarily affects the central nervous system, causing progressive neurodegeneration, including widespread neuronal loss and gliosis. To gain a deeper understanding of the neuropathology associated with GM1 gangliosidosis, we employed single-nucleus RNA sequencing to analyze brain tissues from both GM1 gangliosidosis model mice and control mice. No significant changes in cell proportions were detected between the two groups of animals. Differential expression analysis revealed cell type-specific changes in gene expression in neuronal and glial cells. Functional analysis highlighted the neurodegenerative processes, oxidative phosphorylation, and neuroactive ligand–receptor interactions as the significantly affected pathways. The contribution of the impairment of neurotransmitter system disruption and neuronal circuitry disruption was more important than neuroinflammatory responses to GM1 pathology. In 16-week-old GM1 gangliosidosis mice, no microglial or astrocyte activation or increased expression of innate immunity genes was detected. This suggested that nerve degeneration did not induce the inflammatory response but rather promoted glial cell clearance. Our findings provide a crucial foundation for understanding the cellular and molecular mechanisms of GM1 gangliosidosis, potentially guiding future therapeutic strategies.

## 1. Introduction

GM1 gangliosidosis is a rare lysosomal storage disorder caused by mutations in the β-galactosidase (β-gal) gene (*GLB1*), leading to the accumulation of GM1 gangliosides in the lysosomes of cells in the nervous system. Patients exhibit neurodegenerative symptoms, including cognitive decline and motor dysfunction, which bear striking similarities to the pathological processes observed in neurodegenerative conditions such as Alzheimer’s disease (AD) and Parkinson’s disease (PD) [1]. Although it is known that reduced β-gal activity leads to the accumulation of GM1 gangliosides, the exact mechanisms by which this accumulation causes neurodegeneration and other clinical manifestations remain unclear [2]. Lysosomal accumulation is enhanced throughout disease progression, with an increasing number of swollen lysosomes leading to neural death [3]. Research suggests that these symptoms are linked to mechanisms such as endoplasmic reticulum (ER) stress, calcium homeostasis imbalance, and autophagy dysfunction.

There are currently no approved treatments for GM1 gangliosidosis, but clinical trials using gene therapy (NCT03952637, NCT04713475) and small molecule substrate inhibitors (NCT04221451) are ongoing [4,5]. Although other clinical trials have been initiated in recent years, including the LYS-GM101 gene therapy trial (NCT04273269), a trial testing miglustat as substrate reduction therapy (SRT) (NCT02030015), and one testing umbilical cord-supported stem cell therapy (NCT00654433), these have been terminated before completion [6]. The LYS-GM101 trial, despite some progress relating to safety and preliminary efficacy, did not show significant clinical benefits, and there was limited evidence of improvement in biochemical markers or disease progression [7]. With etiological therapies still in the research phase, the current treatments for GM1 gangliosidosis primarily focus on symptom alleviation. Knowledge of the natural history of this disease is essential for timely diagnosis, facilitating better supportive care, and contextualizing the results of therapeutic trials. Accordingly, a greater understanding of the complex pathogenesis of GM1 gangliosidosis remains urgently needed to foster the development of effective treatments.

GM1 gangliosidosis is one of the most-studied lysosomal storage disorders (LSDs), and understanding its pathobiology offers an opportunity for exploring neurodegenerative processes in the context of lysosomal dysfunction [8]. Both GM1 gangliosidosis and other common neurodegenerative diseases involve the progressive loss of neurons and the disruption of normal cellular processes due to the accumulation of pathological substances. The pathogenesis of these diseases often involves multiple cell types and molecular pathways [9]. Recently, single-cell transcriptome analysis has been extensively applied to characterize neuronal cell populations from the entire brain. Cougnoux et al. used single-cell RNA sequencing (scRNA-seq) to analyze the transcriptomes of individual cells from NPC1-affected cerebella, uncovering significant alterations in gene expression across various cell types, including neurons, astrocytes, and microglia [10]. Additionally, the authors identified specific pathways disrupted in NPC1 and suggested that microglia activation precedes neuronal dysfunction during disease progression. Keren-Shaul et al. identified a unique type of microglia, termed disease-associated microglia (DAM), through scRNA-seq. These cells are characterized by specific gene expression profiles and are activated during neurodegenerative processes, playing a critical role in the neuroinflammatory responses seen in AD [11]. Single-nucleus transcriptomic analysis allows for the identification of cell type-specific responses to lysosomal dysfunction, which is critical for understanding the full spectrum of GM1 gangliosidosis pathology.

Our study employed single-nucleus genomic analysis to explore the disease mechanisms of GM1 gangliosidosis in a mouse model of the disease and identify potential therapeutic targets. By analyzing the molecular characteristics of neurons and other relevant cell types in the brain, we sought to uncover how lysosomal dysfunction induces neuronal damage and understand how these changes relate to the broader mechanisms of neurodegenerative disorders. 

## 2. Results

### 2.1. Single-Nucleus Transcriptome Profiling Identified Cell Populations in the Brains of GM1 Gangliosidosis Mice

**Figure 1:**
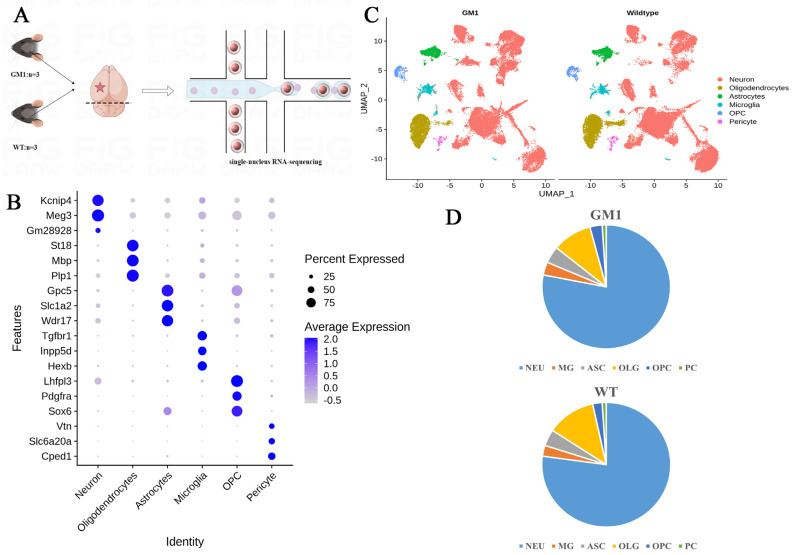
Construction of a single-cell transcriptomic atlas of brain tissue in GM1 mice. (**A**) Single-nucleus RNA sequencing (snRNA-seq) profiling workflow. (**B**) A dot plot demonstrating the classical marker genes used for the identification of a cluster cell type. The dot size reflects the percentage and the color intensity is proportional to the average expression. (**C**) A Uniform Manifold Approximation and Projection (UMAP) plot showing the six major cell types across the brain tissues, based on data from snRNA-seq. (**D**) The proportions of the main cell types in both GM1 and WT brains.

We previously established a mouse model (*Glb1^G455R^^/G455R^*) that mimicked the chronic phenotype of human GM1 gangliosidosis. These mice appear healthy at birth but exhibit ataxia and tremors from 16 weeks of age. To investigate the changes in gene expression in the GM1 gangliosidosis brain at single-cell resolution, we performed single-nucleus RNA sequencing (snRNA-seq) on the brain tissues of *Glb1^G455R/G455R^* (GM1) mice and their sex-matched wild-type (WT) littermates. For this, we isolated cells from the brain hemispheres of three GM1 mice and three WT mice at 16 weeks of age. After stringent quality control, snRNA-seq yielded a total of 69,266 single-nucleus transcriptomic profiles (Figure 1A). The unsupervised clustering of the snRNA-seq dataset resulted in 24 clusters (Supplementary Figure S1A). Based on the known marker genes, six cell types were recognized, including neurons (NEUs), microglia (MG), astrocytes (ASCs), oligodendrocyte precursor cells (OPCs), oligodendrocytes (OLGs), and pericytes (PCs) (Figure 1B) [12,13,14]. The six identified cell types were visualized using a Uniform Manifold Approximation and Projection (UMAP) plot (Figure 1C). All six cell types were shared among mice in both the GM1 and WT groups and were present in similar proportions. The proportion distribution plot in Figure 1D shows that NEUs and OLGs accounted for most of the cells in both groups.

### 2.2. Differential Gene Expression and Functional Analyses Highlighted Abnormal Expression Patterns in Various Cell Types in GM1 Mice

**Figure 2:**
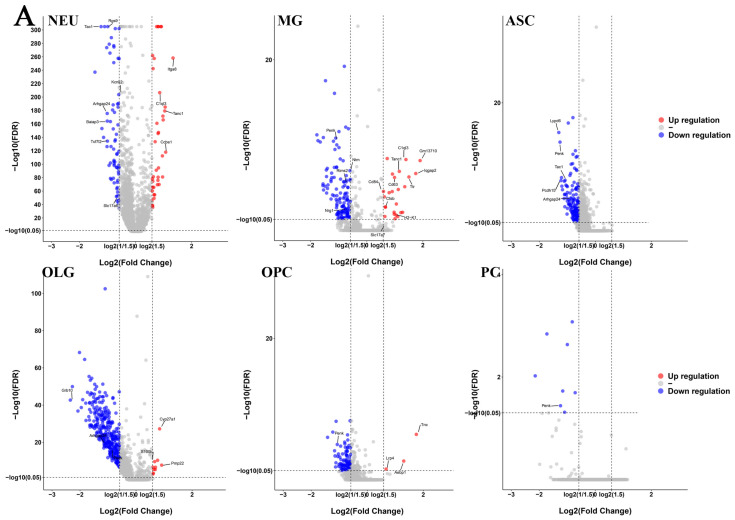

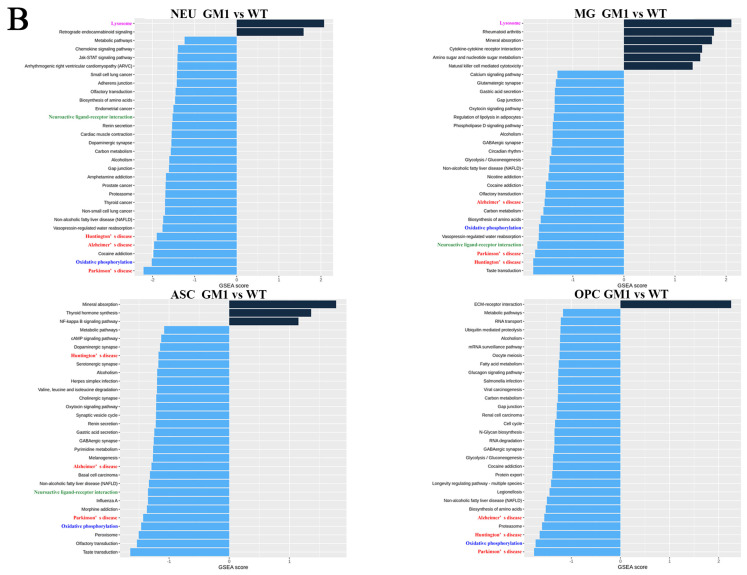
GM1-related changes in gene expression. (**A**) A volcano plot showing –log10 (false discovery rate [FDR]) and logFoldChange (FC) values for the differentially expressed genes (DEGs) of the six cell types. (**B**) A histogram of the Gene Set Enrichment Analysis (GSEA-based pathway enrichment) for the DEGs identified in the major cell types.

To study the effect of GM1 ganglioside accumulation in the mouse brain, we focused on differentially expressed genes (DEGs) on a per-cluster basis between the GM1 group and the WT group (Supplementary Table S1). The quantity and pattern of the DEGs varied among the six cell types (NEUs, MGs, ASCs, OPCs, OLGs, and PCs) (Figure 2A). We found that OLGs had the most DEGs (331), followed by MG, ASCs, NEUs, and OLGs, with 150, 148, 104, and 85 DEGs, respectively. PCs had the least DEGs (8). Because this suggested that the transcriptome of PCs was minimally affected by GM1 ganglioside accumulation, we subsequently concentrated on neuronal- and glial-specific transcripts. We found a greater number of downregulated genes than upregulated ones in GM1 mice. Additionally, in ASCs and PCs, all the DEGs were significantly downregulated. Approximately 38.46% of the DEGs were upregulated and 61.54% were downregulated in NEUs, while most of the DEGs were downregulated in the MG, OLGs, and OPCs. Only the downregulated gene *Penk* was shared across all the cell types. *Penk* encodes a precursor of enkephalins, which are opioid neuropeptides with neuroprotective properties [15]. The upregulation of *Penk* mRNA in the striatum has been reported to exert beneficial effects on behavioral symptoms in Huntington’s disease (HD) in R6/2 mice [16]. *Penk* dysregulation contributes to an imbalance in extracellular neurotransmitter and gliotransmitter homeostasis. Moreover, 119 and 6 DEGs were down- and upregulated, respectively, in at least two cell types, suggesting that cell types share some DEGs. Interestingly, we found that *Arhgap24* was upregulated in MG but downregulated in NEUs, ASCs, and OLGs. ARHGAP24 is a negative regulator of Rac signaling and has been reported to inhibit cell migration by suppressing Rac1 activation [17]. To explore the potential roles of these genes in GM1 gangliosidosis progression, we performed a Gene Set Enrichment Analysis (GSEA) on the DEGs of each cell type. The results indicated that neurodegenerative diseases, including AD, PD, and HD, were the most inhibited biological processes in most cell types in the brains of GM1 mice (Figure 2B). We also observed that the oxidative phosphorylation pathway and the neuroactive ligand–receptor interaction pathway were inhibited in multiple cell types, indicating that metabolism and neurotransmission were disrupted in GM1 mice.

### 2.3. Altered Gene Expression in Neurons of GM1 Mice Induces Neurodegeneration-Related Changes 

**Figure 3:**
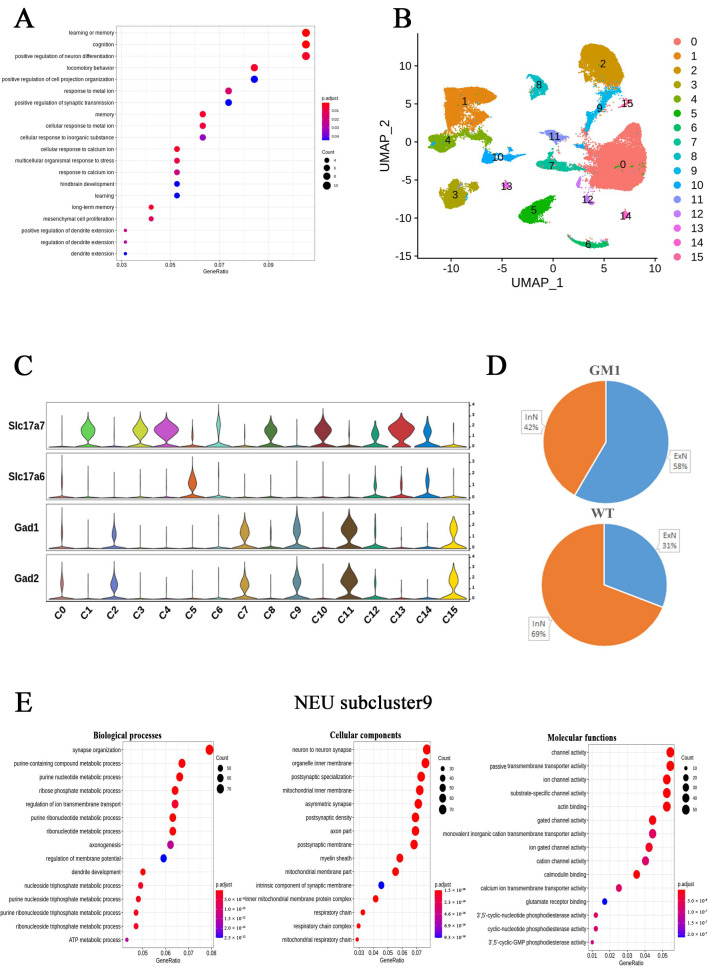
Transcriptional changes in neurons of GM1 gangliosidosis model mice. (**A**) A dot plot showing Gene Ontology (GO) term enrichment based on the differentially expressed genes (DEGs) identified in neurons. (**B**) A UMAP plot showing the NEU subclusters. (**C**) A violin plot showing the expression of marker genes (excitatory neurons [ExNs]: *Slc17a7*, *Slc17a6*; inhibitory neurons [InNs]: *Gad1*, *Gad2*) in neuronal subclusters. (**D**) The proportions of the neuronal subclusters in the brains of GM1 and wild-type (WT) mice. (**E**) A dot plot showing GO term enrichment based on the DEGs identified in subcluster 9.

The proportion distribution plot (Figure 1D) showed that neurons accounted for the majority of cells in both groups of mice. We identified 104 DEGs between GM1 and WT neurons, 64 of which were predominantly downregulated. *Tac1*, *Penk*, *Baiap3*, genes associated with neurotransmission; *Tcf7l2*; and *Rasgrp2*, *Rgs9*, and *Arhgap24*, associated with the regulation of GTPase signaling, were significantly and specifically downregulated in the neurons of GM1 mice. Additionally, Gene Ontology (GO) analysis of all the DEGs in NEUs showed enrichment linked to cellular response to learning or memory, cognition, and locomotory behavior (Figure 3A). These findings supported that GM1 accumulation increased neurodegeneration-associated gene signatures in neurons. Kyoto Encyclopedia of Genes and Genomes (KEGG) pathway enrichment analysis showed that the DEGs in NEUs were associated with the Chemokine signaling pathway (Supplementary Figure S1B).

To explore the changes in neuronal states in GM1 gangliosidosis, we sought to identify the neurons that were affected in GM1 mice. We analyzed 53,782 individual neuronal nuclei and clustered them unsupervised into 16 distinct subclusters after a second round of clustering analysis (Figure 3B). Then, we manually classified these subclusters into excitatory neurons (ExNs; subclusters 1, 3, 4, 5, 6, 8, 10, 12, 13, and 14) and inhibitory neurons (InNs; subclusters 0, 2, 7, 9, 11, and 15) based on the expression of known cell type-specific markers (ExNs: *Slc17a7*, *Slc17a6*; InNs: *Gad1*, *Gad2* [18,19]) (Figure 3C). We found that the proportion of InNs was increased in the GM1 group, whereas that of ExNs was decreased (Figure 3D). However, in each of these subclusters, there was no significant difference in the number of cells between GM1 and WT groups. This might be attributable in part to the relatively limited sample sizes in each group (*n* = 3), resulting in insufficient statistical power. Most of the DEGs in the neuronal subclusters were downregulated, except for subclusters 0, 10, and 14, where the opposite was observed (Supplementary Table S2). 

To investigate the main pathway changes in these subclusters during the progression of GM1 gangliosidosis, we performed a GSEA to identify the DEG pool. Notably, we found that most NEU subclusters showed the enrichment of neurodegeneration-related terms, including “Alzheimer’s disease”, “Huntington’s disease”, and “Parkinson’s disease”. These pathways were significantly downregulated in all InNs, except those of subgroup 0, but were significantly downregulated in only half of the ExNs (including subclusters 1, 3, 4, and 13). Moreover, the “Oxidative phosphorylation” pathway was also downregulated in these clusters. Consistent with these observations, we found that the DEGs were mostly involved in the pathways associated with neurodegenerative diseases based on KEGG pathway analysis. Together, these results preliminarily indicated that relative to ExNs, InNs might be more closely associated with neurodegeneration in GM1 gangliosidosis.

A comparative analysis indicated that subcluster 9 had the greatest number of DEGs among all the InNs, as well as the greatest differences in gene expression. This suggested that subcluster 9 might be the subcluster most affected by GM1 ganglioside accumulation. The results of the GO enrichment analysis of this subcluster showed that in the cellular component (CC) category, the DEGs were associated with terms such as myelin sheath, synapse, and mitochondria. In the molecular function (MF) category, the DEGs were involved in calmodulin binding, ion channel activity, and glutamate receptor binding, among others. For biological process, the terms included synapse organization, purine nucleotide metabolic process, dendrite development, and purine nucleoside triphosphate metabolic process (Figure 3E). These results revealed that ATP metabolism, mitochondrial function, and calcium homeostasis were disrupted in neuronal subcluster 9, which may contribute to the neurodegenerative pathology caused by GM1 accumulation.

### 2.4. Microglia Exert Anti-Inflammatory Functions and Are Involved in the Regulation of Neuronal Death Signaling Pathways in GM1 Mice 

**Figure 4:**
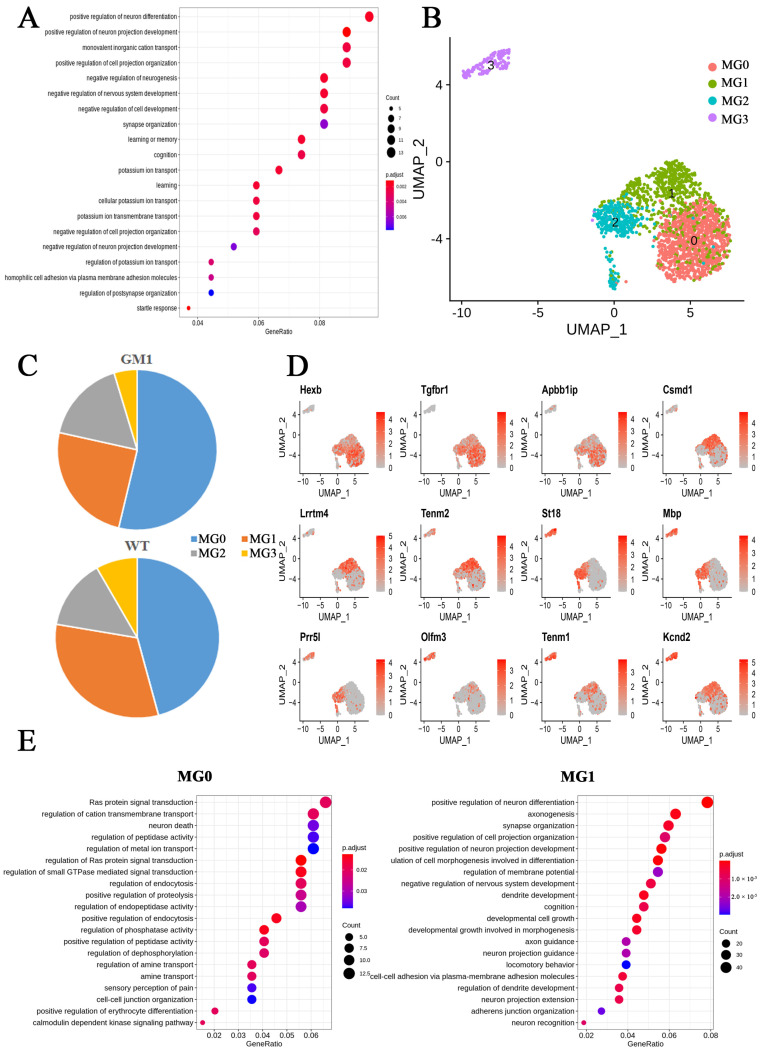
Transcriptional changes in microglia (MG) in GM1 gangliosidosis model mice. (**A**) A dot plot showing GO term enrichment based on the differentially expressed genes (DEGs) identified in microglia. (**B**) UMAP plot showing the MG subclusters. (**C**) The proportions of cells of the MG subclusters in the brains of GM1 and wild-type mice. (**D**) A UMAP plots showing the expression of marker genes in the MG subclusters. (**E**) A dot plot showing GO term enrichment based on the DEGs identified in the MG subclusters.

Microglia abundance was not significantly different between GM1 and WT mice based on the snRNA-seq dataset. The GO analysis of MG was similar to that for NEUs, showing enrichment for the pathways involved in learning or memory and cognition, reflecting that NEU and MG were intimately involved in the process of neurodegeneration (Figure 4A). None of the pathways involved in inflammatory processes were significantly enriched. We obtained a total of 2147 microglial nuclei and further divided them into four subclusters, namely MG0, MG1, MG2, and MG3 (Figure 4B). We observed that microglial marker genes, including *Cx3cr1* and *Siglech*, were highly expressed in all the subclusters, except MG3 (Supplementary Figure S1C). Because MG3 had no continuity with the other subclusters in the UMAP plot and accounted for only 6% of the total microglial population, we excluded this subcluster from the analysis. In the WT group, 45.9% and 31.9% of the microglia were classified into the MG0 and MG1 subclusters, respectively, while the respective proportions in the GM1 group were 53.8% and 24.7% (Figure 4C). These results indicated that microglia exhibited increased differentiation into cells of the MG0 cluster and decreased differentiation into MG1 subcluster cells in the GM1 group. Furthermore, we examined the expression of classic markers for different microglial states. Several microglia states have previously been defined under neurodegenerative conditions, including disease-associated microglia (DAM) found in AD and the microglial neurodegenerative phenotype (MGnD) documented across several disease models [20]. The expression of genes associated with microglial homeostasis (*Hexb*, *Tgfbr1*, *P2ry12*, and *Cx3cr1*) was enriched in the MG0 subcluster [21] (Figure 4D). This subcluster was also the most abundant in both the GM1 and WT groups. Only *Apoe*, a marker gene for DAM and MGnD, was highly expressed in all MG subclusters (Supplementary Figure S1C). The MG1 subcluster showed a high expression of *Csmd1*, *Lrrtm4*, and *Tenm2*, genes with key roles in the development, maintenance, and pruning of synapses in the brain [22,23,24]. Subcluster MG2 exhibited an elevated expression of *St18* and *Mbp*, genes with important functions in myelination [25]. Genes associated with inflammation were not expressed in any of the MG subtypes. These data suggest that microglia-driven neuroinflammation is not a key factor in the early stages of GM1 gangliosidosis. 

The DEGs showed variation among the microglial subclusters. Specifically, more DEGs were downregulated than upregulated in the MG0 subcluster, while the DEGs in the MG1 and MG2 subclusters were mostly upregulated. Only eight DEGs were identified in subcluster MG2, indicating that GM1 accumulation has only a limited effect on cells of this subcluster (Supplementary Table S3). GO analysis highlighted that GM1 ganglioside accumulation induced cell signal pathways in the MG0 subcluster, including Ras protein signal transduction and small GTPase-mediated signal transduction (Figure 4E). In the MG1 subcluster, GM1 ganglioside accumulation induced the expression of genes involved in the regulation of cell projection organization, including those with roles in axonogenesis, synapse organization, and dendrite development. In the MG2 subcluster, GM1 ganglioside accumulation induced the expression of genes with roles in mitochondrion organization and autophagy. Additionally, we found that cells in the MG0 subcluster were involved in the regulation of the neuronal death signaling pathways and the positive regulation of endocytosis (Figure 4E). Collectively, these results indicated that there was high heterogeneity among the four microglial subclusters in terms of functional properties and gene expression profiles in response to GM1 ganglioside accumulation. Our findings further showed that the neurodegeneration caused by GM1 ganglioside accumulation was not primarily due to the activation of inflammation. Moreover, the MG0 subcluster, in which the expression of genes associated with endocytosis was changed, may play an important role in the regulation of neuronal death.

### 2.5. Astrocytes Were Not Activated and Disrupted Synaptic Function Regulation in GM1 Mice 

**Figure 5:**
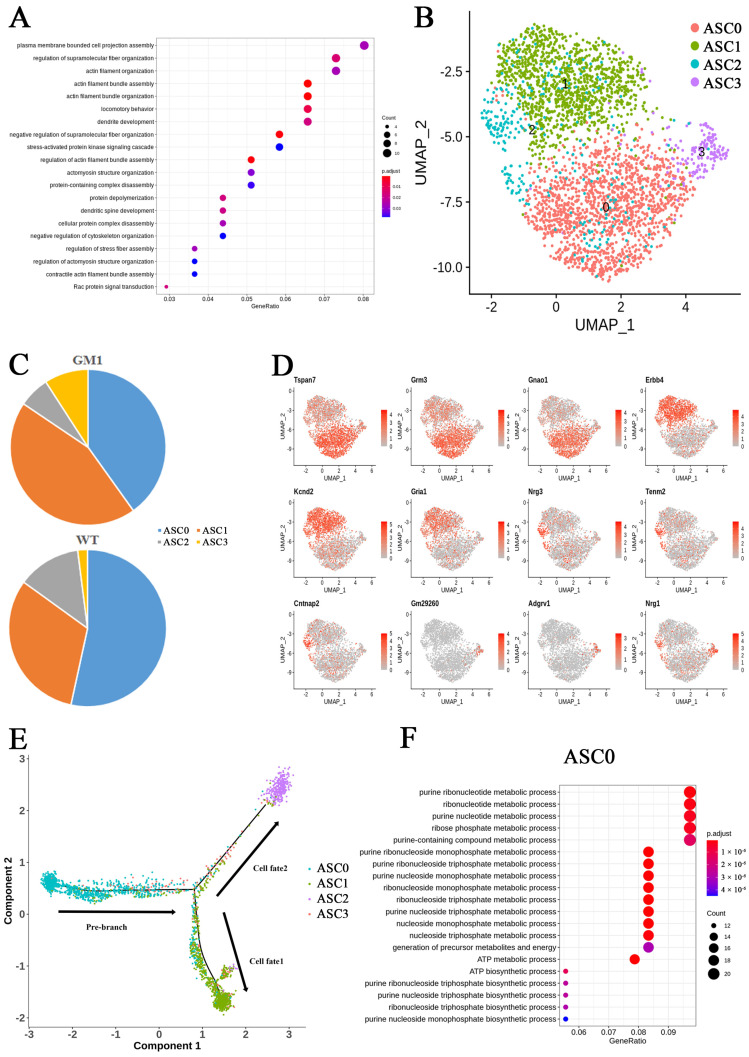
Transcriptional changes in astrocytes (ASCs) in GM1 gangliosidosis model mice. (**A**) A dot plot showing Gene Ontology (GO) term enrichment based on the differentially expressed genes (DEGs) identified in ASCs. (**B**) A UMAP plot showing the ASC subclusters. (**C**) The proportions of the ASC subclusters in the brains of GM1 and wild-type mice. (**D**) A UMAP plots showing the expression of marker genes in the ASC subclusters. (**E**) Pseudo-time showing the relative positioning of ASCs along the trajectory. (**F**) A dot plot showing GO term enrichment based on the DEGs identified in the ASC0 subcluster.

All the DEGs in ASCs were downregulated. We noted a robust downregulation of *Lypd6* and *Penk*, indicative of a state of dysregulation in the ASC neurotransmitter system. GO analysis of the DEGs in ASCs revealed significant enrichment for functional genes with roles in pathways involved in “actin filament bundle assembly”, “locomotory behavior”, and “dendrite development” (Figure 5A). We divided 3025 astrocytes into four subclusters, namely ASC0, ASC1, ASC2, and ASC3 (Figure 5B). A higher proportion of the ASC1 and ASC3 subpopulation was observed in the GM1 brain versus the WT brain, with a lower proportion of the ASC0 and ASC2 subpopulation (Figure 5C). Genes associated with glutamate uptake, such as *Slc1a3*, *Slc1a2*, *Grm3*, *Glul*, and *Grin2c*, were all specifically and highly expressed in the ASC0 subcluster (Supplementary Table S4). Meanwhile, the genes encoding Erbb4 and the glutamate receptor Gria1 were highly expressed in subcluster ASC1. *Nrg1* and *Nrg3*, which interact with Erbb4, were specifically expressed in cells of the ASC2 subcluster. *Nrg1* was highly expressed in both the ASC2 and ASC3 subclusters, while *Nrg3* was only highly expressed in ASC2 (Figure 5D). The expression of genes involved in the positive regulation of synapse assembly was upregulated in the ASC2 subcluster. Nrg1/Erbb4 and Nrg3/Erbb4 signaling have been shown to regulate the inhibitory outputs and excitatory inputs of interneurons in the mouse cerebral cortex, respectively. In rats with spinal cord injury, intrathecal treatment with Nrg1 converted reactive astrocytes to oligodendrocyte lineage cells, inhibited astrogliosis, promoted remyelination, protected axons, and eventually improved Basso, Beattie, and Bresnahan scores [26]. To further investigate the differentiation trajectory of ASCs, we used pseudo-time analysis to mimic their specific differentiation processes. We found that subcluster ASC0 was situated at the beginning of the pseudo-time trajectory and gradually bifurcated into two distinct branches, in which the cells differentiated toward ASC1 or ASC2 lineages (Figure 5E).

GO analysis of the DEGs in ASCs revealed that, in the BP category, cells in the ASC0 subcluster were associated with ATP metabolic process, indicative of the presence of energy metabolism disorder in these cells (Figure 5F). The ASC1 subcluster was associated with developmental cell growth, while the ASC2 and ASC3 subclusters were associated with synapse organization in GM1 mice (Supplementary Figure S1D). In conclusion, GM1 ganglioside accumulation may impair ATP metabolism and synaptic function regulation in astrocytes. None of the ASC subtypes featured the neurodegenerative disease-associated A1 or A2 phenotype markers, which suggested that ASCs had not converted to an A1 or A2 phenotype. 

### 2.6. OLGs and OPCs Displayed Distinct Responses in GM1 Mice

**Figure 6:**
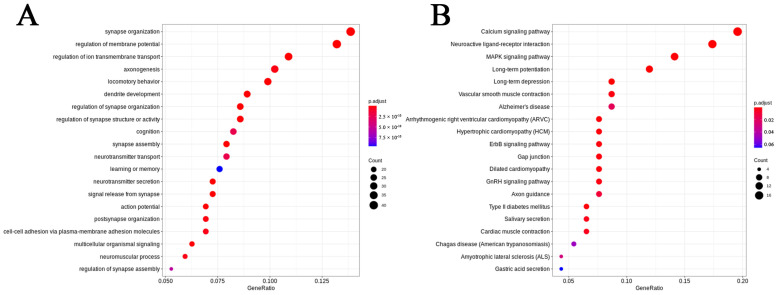

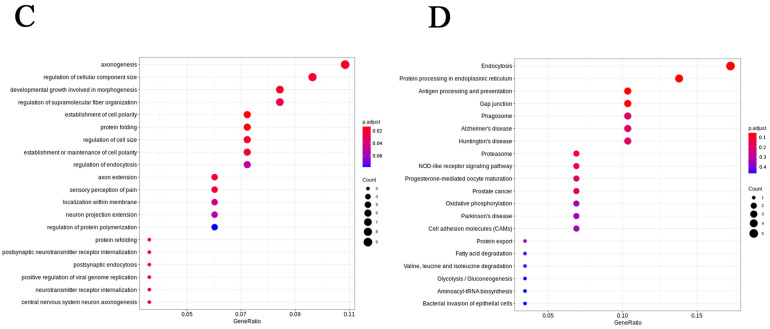
Transcriptional changes in oligodendroglial lineage cells in GM1 gangliosidosis model mice. (**A**) A dot plot showing Gene Ontology (GO) term enrichment based on the differentially expressed genes (DEGs) identified in oligodendrocytes (OLGs). (**B**) A dot plot showing GO term enrichment based on the DEGs identified in oligodendrocyte progenitor cells (OPCs). (**C**) Kyoto Encyclopedia of Genes and Genomes (KEGG) pathways associated with the DEGs in OLGs. (**D**) KEGG pathways associated with the DEGs in the OPCs.

We obtained a total of 7745 OLG nuclei and 1886 OPC nuclei. Compared with the WT group, the proportion of OLGs was decreased in the GM1 group, whereas that of OPCs was increased. Only 15 downregulated DEGs were shared between OLGs and OPCs, indicative of distinct responses to GM1 ganglioside accumulation between these two cell types (Supplementary Table S1). There were more DEGs in the OLGs than in the OPCs, which indicated that the former were more sensitive to GM1 ganglioside accumulation. The most significant DEG in the OPCs was *Tnc*, which was upregulated 3-fold. It has been reported that Tnc has an inhibitory effect on OPC migration and differentiation as well as on myelin formation and remyelination [27]. In OLGs, the most significant DEG was *Grb10*, exhibiting a 5-fold downregulation. Grb10 is the binding partner for several transmembrane tyrosine kinase receptors, including insulin receptor (IR) and insulin-like growth factor-1 receptor (IGF1-R) [28]. Additionally, high Grb10 expression was reported to be harmful to cognitive function in diabetic rats [29]. GO analysis of the DEGs revealed that they were enriched in the biological processes of axonogenesis in both the OLGs and OPCs (Figure 6A,B). Additionally, the DEGs were enriched in the biological processes of regulation of ion transmembrane transport in the OLGs and the regulation of cellular component size in the OPCs (Figure 6C,D). These results showed that GM1 ganglioside accumulation elicited differential responses in OLGs and OPCs.

### 2.7. Changes in Intercellular Communication in the CNS Revealed the Microenvironment in the GM1 Mouse Brain

**Figure 7:**
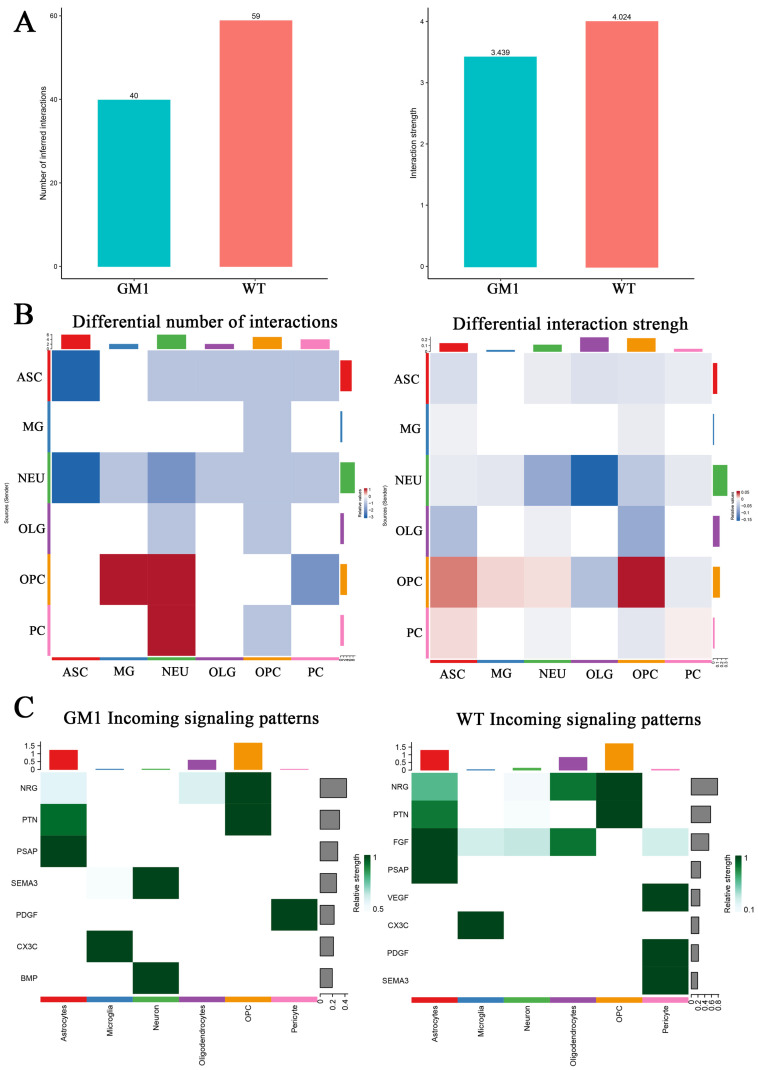
Changes in intercellular communication in GM1 gangliosidosis model mice. (**A**) The number and strength of ligand–receptor interactions in the GM1 and wild-type (WT) groups. (**B**) Heatmaps of the interactions change between GM1 and WT groups. (**C**) Heatmaps of the incoming signaling patterns in the GM1 and WT groups.

Comparing the interaction among the different cell types in the CNS microenvironment can help to understand the pathogenesis of GM1 gangliosidosis as well as identify potential therapeutic targets for the disease. Here, we constructed an intercellular communication network of different cell-type subpopulations based on ligand–receptor pairs. In the GM1 group, the strength of cell–cell interactions was decreased in most cell types (Figure 7A). ASCs had decreased interactions with NEUs and themselves; NEUs had decreased interaction strength with OLGs, PCs and themselves; OPCs had increased interactions with NEUs and MG, and increased interaction strength with themselves. The interactions associated with MG were less variable in GM1 gangliosidosis (Figure 7B). 

NEU was the cell type with the most pronounced decrease in cell–cell communication in GM1 gangliosidosis. We further compared the information flow from NEUs for each signaling pathway between the GM1 and WT groups. Compared with the WT group, incoming interactions from other cells to NEUs relating to neuroprotection (Nrg1/Erbb4 and Fgf1/Fgfr2) were decreased in the GM1 group, whereas those related to axonogenesis (Sema3d/Pxlna4) and synapse formation (Bmp6/Bmpr1) were increased (Figure 7C). Our analysis suggested that the alteration in intercellular communication might be a previously underestimated aspect of GM1 gangliosidosis pathogenesis and one that merits further exploration.

## 3. Discussion

The unquestionable cause of GM1 gangliosidosis is the attenuation or absence of β-gal activity. However, how GM1 ganglioside accumulation leads to neurodegeneration remains unclear. Comparisons of transcriptome data between controls and disease models can help gain insight into these processes. In this study, we used snRNA-seq for the first time to analyze the molecular characteristics of neurons and glial cells in a GM1 gangliosidosis mouse model. We compared brain snRNA-seq data between *Glb1^G455R/G455R^* (GM1) and *Glb1*^+/+^ mice (WT) at 16 weeks of age. We focused on the brain, given that GM1 gangliosides primarily accumulate in the CNS, and the resulting neurodegeneration is a cardinal symptom of this disorder. 

First, we found that GM1 ganglioside accumulation induced cell-specific responses in the brains of mice at the single-cell level. Most of the DEGs were downregulated in the glial cell cluster, including MG, ASCs, OLGs, and OPCs. NEUs had the most upregulated genes of all the cell types (40) and also had 64 downregulated genes. We noted that the DEGs in all the cell types between the GM1 and WT groups were enriched in the GO terms of learning or memory, cognition, and locomotor behavior. The dysregulated expression of genes related to these terms provides evidence that GM1 ganglioside accumulation leads to neurodegeneration in the brains of 16-week-old GM1 mice.

We explored the potential pathways through which these DEGs influence the pathogenesis of GM1 gangliosidosis. The mechanisms of this disease are thought to include UPR-mediated apoptosis, neurotransmission, autophagy, neurotrophic factor activity, and neuroinflammation [30]. Here, we found that the DEGs converged on mechanisms associated with synaptic structure and synaptic function regulation. GO enrichment analysis identified the terms “positive regulation of cell projection organization”, “positive regulation of synaptic transmission”, and “positive regulation of dendrite extension” as being significantly associated with the DEGs in NEUs. The DEGs in MG were significantly enriched in the GO terms “positive regulation of neuron projection development” and “synapse organization”. Additionally, the DEGs in OLGs were significantly enriched in the GO terms “synapse organization”, “axonogenesis”, and “dendrite development”, while those in OPCs were significantly associated with the GO terms “axonogenesis” and “axon extension”. Given the key nutritive support provided by OLGs and OPCs during neuron development [31,32], the dysregulation of genes associated with axonogenesis may lead to futile axonogenesis in the brains of GM1 mice. Meanwhile, attenuated β-gal activity leads to increased GM1 ganglioside and cholesterol accumulation at the synaptosomal membrane, and, consequently, the emergence of detergent-resistant lipid microdomains [33]. Indeed, a growing body of research has documented that neuronal death due to synaptic dysregulation contributes to neurodegeneration [34,35]. Moreover, the disruption of neuronal circuitry affects neurotransmission, which may also contribute to neuronal loss. It has been reported that excitatory synaptic dysregulation elicits excitotoxicity, which contributes to neuronal death following acute brain injury such as stroke or trauma [36].

We observed that neuroactive ligand–receptor interaction emerged as a common pathway enriched in most cell types based on GSEA. Impaired neurotransmitter release from presynaptic sites has been observed in mature neurons derived from GM1 gangliosidosis induced pluripotent stem cells (iPSCs) and model mice [37,38]. Meanwhile, we found that the expression of neurotransmitter-associated genes, such as *Tac1*, *Penk*, and *Baiap3*, was downregulated in the brain cells of GM1 mice. *Tac1* encodes endogenous substance P, which can reportedly prevent the delayed degeneration of dopaminergic neurons after stroke [39]. *Penk* encodes preproenkephalin, the levels of which are reduced in several neurodegenerative diseases. In the early stages of HD and PD, increased expression of pre-enkephalin can attenuate disease symptoms [16,40]. *Baiap3* encodes brain-specific angiogenesis inhibitor 1-associated protein 3. In models where *Baiap3* is downregulated, both in vitro and in vivo, 5-HT exocytosis and levels in the synapse are reduced, resulting in defective post-synaptic neurotransmission [41]. Singer et al. reported that changes in synaptic glycolipid composition and the associated impairment of neurotransmitter uptake lead to neuronal dysfunction [38]. The dysregulation of excitatory and inhibitory neurotransmission in the brain also promotes neuronal death [42]. Excitotoxicity-mediated neuronal cell death, which has been observed in ischemia and neurodegenerative diseases, is the consequence of imbalances in glutamatergic neurotransmission. Different studies have shown that genetic mutations affecting neurotransmitter systems cause synaptic dysfunction and neuronal death in neurodegenerative disorders [43]. In conclusion, our study provides valuable evidence supporting that neuronal circuit defects may be a contributing factor to early neurodegeneration in GM1 gangliosidosis. 

The neuroinflammatory response is another common feature of neurodegeneration. Jeyakumar et al. demonstrated that in Sandhoff and GM1 mice, microglial activation and macrophage infiltration were observed in almost every region of the brain, accompanied by significant astrogliosis [44]. However, we did not find strong evidence of an overt inflammatory response in GM1 mice at 16 weeks of age. We observed neither an increase in the numbers of activated astrocytes and proinflammatory microglia nor an increase in the expression of genes related to proinflammatory cytokines in the brains of GM1 mice, based on our subcluster analysis of single-nucleus transcriptomic data. Additionally, none of the cell types displayed pathway enrichments linked to immune system development and signaling involved in the regulation of immune responses. Our previous study showed that the GM1 model mice are still in the early stages of the disease at 16 weeks of age [45]. Given that GM1 ganglioside accumulation and neuromotor dysfunction had occurred in 16-week-old GM1 mice, we expected to observe early transcriptome changes before the onset of massive neural death. Unexpectedly, although GM1 ganglioside accumulation is present at this age, the level might not be severe to induce significant inflammation at this age. However, we did observe multiple early transcriptome changes in MG. GSEA revealed that lysosome-related genes were upregulated in both NEUs and MG. Meanwhile, the upregulated DEGs in MG were enriched in the GO term “exocytosis”. The promotion of lysosomal exocytosis has been proposed as a minor mechanism involved in the clearance of stored cholesterol [10,46]. This may indicate that, in the GM1 mouse brain, MG and NEUs clear GM1 ganglioside and other substrates in the lysosome by enhancing lysosomal function and promoting lysosomal exocytosis. Initial metabolic changes resulting from GM1 accumulation in lysosomes may first occur in NEUs and MG. Our current data support that early microglial-specific responses may be neuroprotective in GM1 gangliosidosis and contribute to the clearance of degenerating neurons. Taken together, these findings suggest that GM1 ganglioside accumulation in the early stages of GM1 gangliosidosis did not provoke severe inflammation in GM1 mice, and that GM1 ganglioside accumulation may lead to an anti-inflammatory response in the early stages of GM1 gangliosidosis. 

Brain cells form and maintain an interconnected microenvironment, which is essential for efficient neuronal function in the brain. Deciphering ligand–receptor-mediated interactions can help elucidate the cell-to-cell communication that occurs during GM1 ganglioside accumulation, thereby providing novel options for the treatment of neurodegenerative diseases. In this work, our intercellular interaction analysis demonstrated that the Fgf1/Fgfr2 interaction was reduced between NEUs and ASCs or OLGs in GM1 mice. The FGF signaling pathway plays an important role in cell survival and proliferation and is involved in modulating oligodendrocyte differentiation and myelination [47]. Wen-Cheng Huang et al. and Jiawei Li et al. showed that endogenous FGF1 levels decrease in the rat spinal cord following injury. Augmenting its expression, through virally mediated- gene transfer, promotes functional recovery via neuroprotection, axon regeneration and remyelination [48,49]. Additionally, FGFR1/FGFR2 double-knockout mice exhibit defects in myelination [50]. Thus, we speculate that the absence of Fgf1/Fgfr2 in GM1 mice may limit the myelination ability of OLGs.

Finally, our study had several limitations. One was the relatively small sample size, which reduced the statistical power of the data. Another was the age of the mice. At 16 weeks of age, GM1 mice had mild disease symptoms, and may not accurately reflect the full complexity of GM1 gangliosidosis pathology. Finally, single-nucleus sequencing might not capture low-abundance transcripts, potentially leading to small biases in the data. 

In summary, we drew a panoramic picture of cell-specific responses in the GM1 gangliosidosis mouse model, which might provide fundamental support for exploring the mechanisms underlying the neurodegeneration induced by GM1 ganglioside accumulation in the brain. Based on this atlas, our work supports that GM1 ganglioside accumulation leads to neurodegeneration in 16-week-old GM1 model mice, primarily by inducing neurotransmitter dysregulation and impairing synaptogenesis in the brain.

## 4. Materials and Methods

### 4.1. Animals

*Glb1^G455R^* knock-in (KI) mice (in a C57BL/6N background) were generated by Cyagen Biosciences (www.cyagen.com) using CRISPR/Cas9. The genotypes of GM1 gangliosidosis model mice (KI/KI, GM1) and their age-matched WT littermates (+/+, WT) were confirmed by amplifying genomic DNA obtained from the tail of the mice. The mice were housed in specific-pathogen-free conditions with a 12 h/12 h light/dark cycle and had free access to food and water. At 16 weeks, the animals were anesthetized using 1% sodium pentobarbital (50 mg/kg) and sacrificed by cervical dislocation. Brain hemispheres were harvested and quickly frozen and stored in liquid nitrogen. All animal procedures were approved by the Institutional Animal Care and Use Committee of Guangzhou Medical University and were performed in accordance with institutional guidelines.

### 4.2. Single-Nucleus RNA Sequencing

To isolate nuclei, three mouse brains per group were quickly placed in lysis buffer and ground to a liquid using a tissue homogenizer. The tissue lysate was passed through a 40-μm cell sieve to remove impurities, transferred to a new 2 mL Eppendorf tube, and centrifuged at 500× *g* for 5 min at 4 °C to obtain the precipitate. Next, PB1, PB2, and PB3 solutions were sequentially added to the precipitate, with the nuclei located at the interface of the PB2 and PB3 solutions. Finally, the nuclei were resuspended by blowing into a 5-μL NB solution. Nuclei were counted with a cell counter (Thermo Fisher, Waltham, MA, USA). SnRNA-seq libraries were constructed using a Chromium Single Cell 3′ Library and Gel Bead Kit v3 (10× Genomics, Pleasanton, CA, USA) according to the manufacturer’s instructions. Nuclei were immediately loaded onto a Chromium Single Cell Processor (10× Genomics) for the barcoding of RNA from single nuclei. The libraries were sequenced on a NovaSeq 6000 system (Illumina, San Diego, CA, USA).

### 4.3. Data Processing and Quality Control

After sequencing, Cell Ranger 5.0.0 software from 10× Genomics was used for data alignment, gene quantification, and cell identification. Illumina sequencing data in FASTQ format were aligned to the mouse genome, version GRCm38, using the STAR algorithm [51]. This output was then imported into the Seurat (v3.2.0) R toolkit for quality control and downstream analysis of the snRNA-seq data [52]. All functions were run with the default parameters unless otherwise specified. To ensure data reliability, the following filtering metrics were applied: (1) Nuclei with a percentage of expressed mitochondrial genes greater than 20% were removed to exclude low-activity cells; (2) nuclei with UMI counts greater than 10,000 were removed to exclude doublet-like cells; and (3) nuclei with fewer than 200 or more than 5000 detected genes were excluded; each gene had to have at least one UMI aligned in at least three nuclei.

### 4.4. Clustering and Cell Type Identification

The data were normalized (NormalizeData function in Seurat) to extract a subset of variable genes. Variable genes were identified while controlling for the strong relationship between variability and average expression. A total of 2000 highly variable genes were then selected to integrate all sequencing libraries using the ‘FindIntegrationAnchors’ and ‘IntegrateData’ functions, followed by technical noise regression [53]. Principal component analysis (PCA) was conducted on the integrated output matrix and the data were reduced to the top 30 PCA components after scaling. Cells were clustered using graph-based clustering of the PCs with the Louvain method after computing a shared nearest-neighbor graph [54]. For each cluster, the Wilcoxon rank-sum test was used to find significant DEGs relative to the remaining clusters. SingleR [55] and known marker genes (mainly based on Cell Marker 2.0 databases) were used for cell-type identification. The clusters were visualized on a 2D map produced with UMAP. To further refine the analysis, the same procedure (scaling, dimensionality reduction, and clustering) was applied to the specific set of data (restricted to one type of cell) for subcluster analysis within each cell type. 

### 4.5. Differential Gene Expression and Pathway Analysis

Within each cluster, differential expression analysis was performed between the GM1 and WT groups using the Wilcoxon algorithm from the Seurat package, with the adjusted *p*-value determined using the Bonferroni correction. Thresholds of q_adjust < 0.05 and FC > 1.5/FC < 0.66 were used to filter DEGs. Additionally, the DEGs had to be expressed in at least 10% of cells from one of the two groups for that cell type. 

DEG functional enrichment analysis was performed using the GO [56] and KEGG [57] databases via the clusterProfiler package in R (v. 3.14.0), applying a hypergeometric test and correcting for multiple hypotheses using the FDR method. Additionally, GSEA was conducted with the clusterProfiler package, with significance set at FDR ≤ 0.25 and *p* ≤ 0.05.

### 4.6. Cell–Cell Communication Analysis

Cell–cell communication analysis based on the snRNA-seq data was conducted using CellChat (v.0.5.0). To identify significant cell–cell interactions, permutation tests were performed between two cell types mediated by a specific ligand–receptor pair based on the mean ligand gene expression from one cell type and the corresponding receptor from another cell type, with *p* < 0.01 determining statistical significance. The number of significant ligand–receptor pairs represented the weights of the edges between each pair of cell types. Communication networks, including signaling pathways and ligand–receptor (L–R) pairs, were calculated separately for the GM1 and WT groups, and then the network differences were compared between the two conditions. Given that interaction number and strength were key factors, the ‘compareInteractions’ function was used to obtain differences in the whole network interaction number and strength. At the L–R pair level, dysfunctional L–R pairs were identified using differential expression analysis with the ‘identify Over Expressed Genes’ and ‘net Mapping DEG’ functions, detecting upregulated and downregulated L–R pairs.

## Data Availability

If necessary, further information is available from the corresponding author on reasonable request.

## Supplementary Materials

The following supporting information can be downloaded at: https://www.mdpi.com/article/10.3390/ijms25179712/s1.
