# Supplementary material for: Insights into the Pathobiology of GM1 Gangliosidosis from Single-Nucleus Transcriptomic Analysis of CNS Cells in a Mouse Model
Source: Int J Mol Sci. 2024 Sep 8;25(17):9712. doi: 10.3390/ijms25179712 (PMC11395632; doi:10.3390/ijms25179712)
Supplement: Supplementary file 1 [file ijms-25-09712-s001.zip › Supplementary Figure S1aRevised.pdf]

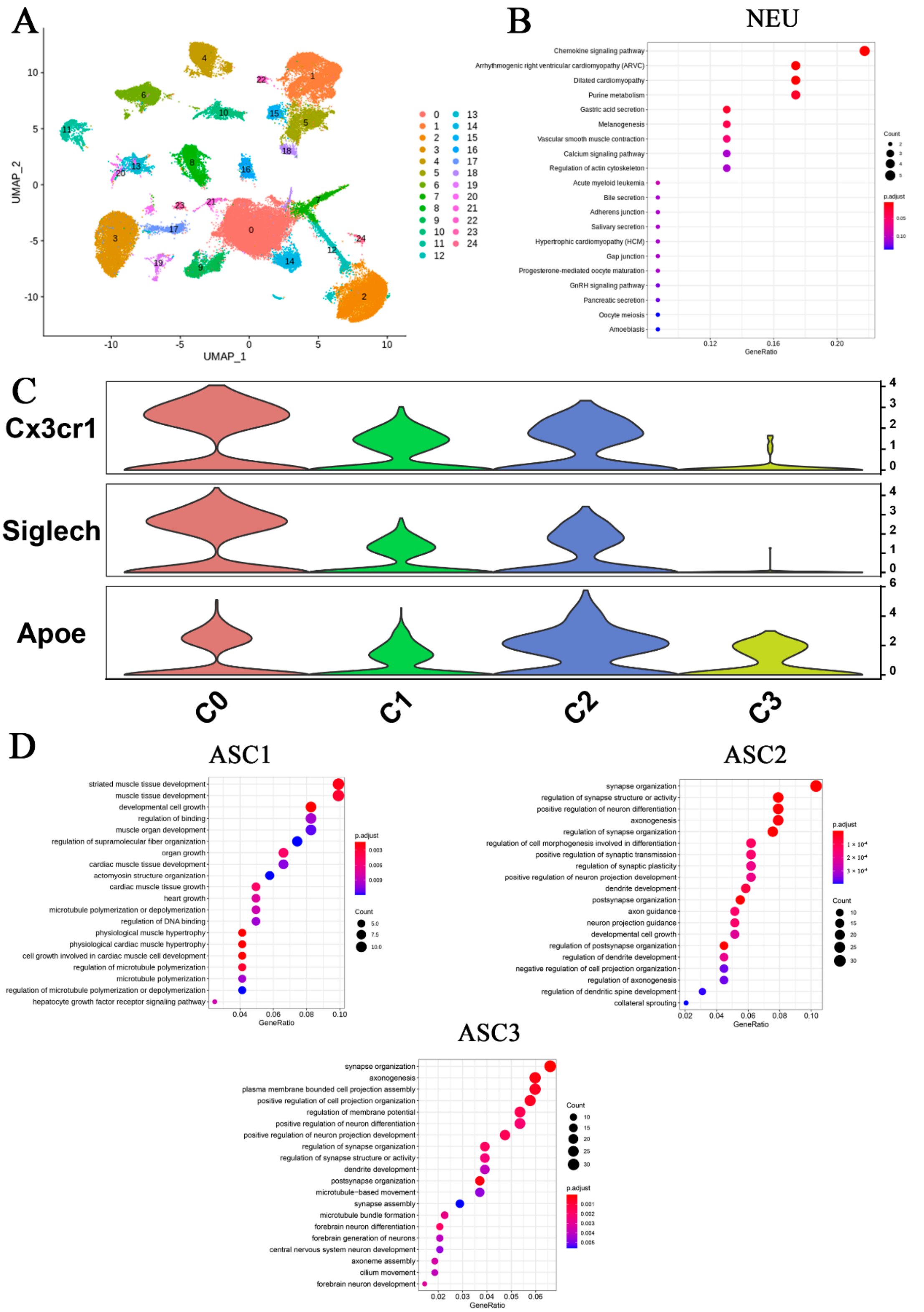

Supplementary Figure S1a: (**a**) A Uniform Manifold Approximation and Projection (UMAP) plot showing 24 clusters across the brain tissues based on data from snRNA-seq. (**b**) Kyoto Encyclopedia of Genes and Genomes (KEGG) pathways associated with the DEGs in NEUs. (**c**) A violin plot showing the expression of microglial marker genes （Cx3cr1, Siglech and Apoe）in MG subclusters. (**d**) A dot plot showing GO term enrichment based on the differentially expressed genes (DEGs) identified in the ASC subclusters.
